# Metaprop: a Stata command to perform meta-analysis of binomial data

**DOI:** 10.1186/2049-3258-72-39

**Published:** 2014-11-10

**Authors:** Victoria N Nyaga, Marc Arbyn, Marc Aerts

**Affiliations:** Unit of Cancer Epidemiology, Scientific Institute of Public Health, Juliette Wytsmanstraat 14, 1050 Brussels, Belgium; Center for Statistics, Hasselt University, Agoralaan Building D, 3590 Diepenbeek, Belgium

**Keywords:** Meta-analysis, Stata, Binomial, Logistic-normal, Confidence intervals, Freeman-Tukey double arcsine transformation

## Abstract

**Background:**

Meta-analyses have become an essential tool in synthesizing evidence on clinical and epidemiological questions derived from a multitude of similar studies assessing the particular issue. Appropriate and accessible statistical software is needed to produce the summary statistic of interest.

**Methods:**

*Metaprop* is a statistical program implemented to perform meta-analyses of proportions in Stata. It builds further on the existing Stata procedure *metan* which is typically used to pool effects (risk ratios, odds ratios, differences of risks or means) but which is also used to pool proportions. *Metaprop* implements procedures which are specific to binomial data and allows computation of exact binomial and score test-based confidence intervals. It provides appropriate methods for dealing with proportions close to or at the margins where the normal approximation procedures often break down, by use of the binomial distribution to model the within-study variability or by allowing Freeman-Tukey double arcsine transformation to stabilize the variances. *Metaprop* was applied on two published meta-analyses: 1) prevalence of HPV-infection in women with a Pap smear showing ASC-US; 2) cure rate after treatment for cervical precancer using cold coagulation.

**Results:**

The first meta-analysis showed a pooled HPV-prevalence of 43% (95% CI: 38%-48%). In the second meta-analysis, the pooled percentage of cured women was 94% (95% CI: 86%-97%).

**Conclusion:**

By using *metaprop*, no studies with 0% or 100% proportions were excluded from the meta-analysis. Furthermore, study specific and pooled confidence intervals always were within admissible values, contrary to the original publication, where *metan* was used.

**Electronic supplementary material:**

The online version of this article (doi:10.1186/2049-3258-72-39) contains supplementary material, which is available to authorized users.

## Background

Meta-analyses combine information from multiple studies in order to derive an average estimate. Different meta-analysis procedures exist depending on the statistic to be reported. Examples of statistics of interest include association measures such as risk difference, risk ratio, odds ratio, difference in means, or simply one-dimensional binomial or continuous measures such as proportions or means.

There are three important aspects in meta-analysis: a) the analysis framework, b) the model and c) the choice of the method to estimate the heterogeneity parameter. These aspects interact with each other. A meta-analyst has a choice between the fixed- and random-effects model.

In the fixed-effects model, it is assumed that the parameter of interest is identical across studies and the difference between the observed proportion and the mean is only due to sampling error. In the random-effects model, the observed difference between the proportions and the mean cannot be entirely attributed to sampling error and other factors such as differences in study population, study designs, etc. could also contribute. Each study estimates a different parameter, and the pooled estimate describes the mean of the distribution of the estimated parameters. The variance parameter describes the heterogeneity among the studies and in the case where the variance is zero, this model simply reduces to the fixed-effects model.

There are three frameworks in modeling of binomial data. The most popular framework uses approximation to the normal distribution by use of transformations and is known as the approximate likelihood approach [1, 2]. Some of the common transformations include the logit and the arcsine [3]. Some of the reasons why this approach is popular include lower level of required statistical expertise, faster computations and availability of software to carry out the analysis.

The second approach recognises the true nature of the data and is known as the exact likelihood approach. In this framework, the special relationship between the mean and the variance as characterised by binomial data is captured by the binomial distribution [4]. The beta-binomial distribution [5] can be used to fit a random-effects model such that the beta distribution describes the distribution of the varying binomial parameters. While it is possible to perform computations to estimate the parameters of the binomial model, most common statistical software lacks function to fit the beta-binomial model and therefore, this approach is the least popular. The WinBUGS software, a software package for Bayesian statistics, has the capability to perform such analyses. Other software e.g R and SAS (PROC NLMIXED) can also be used, but extensive programming is required.

The third approach is a compromise between approximate and exact likelihood. In the first stage, the data is modeled using the binomial distribution. In the second stage, the normal distribution is used after the logit transformation to model the heterogeneity among the studies. This is an emerging approach and is often recommended by statisticians [4]. Most statistical software including Stata(melogit), R, SAS (PROC NLMIXED) have the capability to perform such analyses.

There are three popular methods to estimate the parameters. The non-iterative method popularised byDersimonian and Laird [6]. The other two methods are the maximum likelihood (ML) and restricted maximum likelihood (REML) method. For random-effects model, the REML method is preferred because ML leads to underestimation of the variance parameter. For generalized linear mixed models [2, 7, 8] under which models for binomial data falls, the REML method is not used due to intensive computation of high-dimension integrations of the random-effects and as a result most software estimate the heterogeneity parameter using ML methods. The procedure proposed by Dersimonian and Laird is efficient for the mean but not the heterogeneity parameter [9].

Various procedures to perform meta-analysis have been implemented in the Stata command *metan*[10]. In *metan*, the confidence intervals are calculated using the normal distribution based on the asymptotic variance. For proportions such intervals may contain inadmissible values especially when the statistic is near the boundary. Furthermore, computation of confidence intervals is not possible when the statistic is on the boundary, as the estimated standard error is set to zero and as a consequence, the *metan* command automatically excludes studies with proportion equal to 0 or 1 from the calculation of the pooled estimate.

Tests of significance on the pooled proportion typically rely on normal probabilities. Proportions ($p=\frac{r}{n}$) are binomial and the normal distribution is a good approximation of the binomial distribution if *n* is large enough and *p* is not close to the margins [11]. When *n* is small and/or *p* is near the margins, the test statistic may not be approximately normally distributed due to its skewness and discreteness. To make the normal distribution assumptions more applicable to significance testing, several transformations have been suggested. Freeman and Tukey [12] presented a double arcsine transformation to stabilize the variance.

We have developed *metaprop*, a new program in Stata to perform meta-analyses of binomial data to supplement the *metan* command, which is typically used to pool associations. *metaprop* builds further on the *metan* procedure. It allows computation of 95% confidence intervals using the score statistic and the exact binomial method and incorporates the Freeman-Tukey double arcsine transformation of proportions. The program also allows the within-study variability be modelled using the binomial distribution. This article presents a general overview of the program to serve as a starting point for users interested in performing meta-analysis of proportions in Stata software.

## Methods

A detailed description of various statistical procedures to perform meta-analysis which can be performed with *metan* can be found elsewhere [10]. In this article, we present procedures specific to pooling of binomial data including methods of computation of the confidence intervals, continuity correct and the Freeman-Tukey transformation. Table 1 summarises the characteristics of the procedures presented.

**Table 1:** **Summary of the procedures available in metaprop**

Option in metaprop	Description	Strength	Remarks
cimethod (score)	Computes the study specific confidence intervals using the score method.	Study specific intervals always yield admissible values (within the limits of 0 and 1).	The Wald confidence intervals for the pooled estimate could be inadmissible if study specific estimates are on or close to the margin.
		The coverage probability of the study specific confidence intervals are close to the nominal level.	
cimethod (exact)	Computes the study specific confidence intervals using exact method	Study specific intervals always yield admissible values	More conservative method and therefore study specific confidence intervals tend to be too wide.
			The Wald confidence intervals for the pooled estimate could be inadmissible if study specific estimates are on or close to the margin.
ftt	Performs the Freeman-tukey double arcsine transformation, computes the weighted pooled estimate and performs the back-transformation on the pooled estimate.	The confidence intervals for the pooled estimate are always admissible. Test of significance based on Normal approximation more applicable than without the transformation.	The procedure could break-down in case of extremely sparse data.
logit	Uses the Binomial distribution to model the within-study variability.	The confidence intervals for the study-specific estimate and pooled estimate are always admissible.	Requires *metaprop_one* available for Stata 13 or later versions.
			It is an iterative procedure and therefore it requires more computational time than non-iterative procedures.

### Confidence intervals for the individual studies

Two types of confidence intervals for the study specific proportions have been implemented. Throughout the text, for study *i*, *r*_*i*_ denotes the number of observations with a certain characteristic, *n*_*i*_ is the total number of observations, $p_{i}=\frac{r_{i}}{n_{i}}$ is the observed proportion, k is the total number of studies in the meta-analysis, and *1* - *α* refers to the selected level of confidence.

#### Exact confidence intervals

The exact or Clopper-Pearson [13] confidence limits for a binomial proportion are constructed by inverting the equal-tailed test based on the binomial distribution.

The interval for the *i*^*th*^study is [ *L*_*i*_, *U*_*i*_] with *L*_*i*_ and *U*_*i*_ as the solutions in *p*_*i*_ to the equations;

$$\begin{array}{c} P\left(X_{i}\geq r_{i}\right)=\frac{\alpha }{2}\;\text{and}\;P\left(X_{i}\leq r_{i}\right)=\frac{\alpha }{2}\;\text{for}\;X_{i}=0,1,\mathrm{..}r_{i},\ldots ,n_{i}. \end{array}$$

The lower endpoint is the $\frac{\alpha }{2}$ quantile of a beta distribution; Beta(*x*_*i*_,*n*_*i*_-*x*_*i*_+1), and the upper endpoint is the $1-\frac{\alpha }{2}$ quantile of a beta distribution; Beta(*x*_*i*_+ 1,*n*_*i*_-*x*_*i*_) [14]. Since the binomial distribution is discrete, the coverage probability of the exact intervals is not exactly (1- *α*) but at least (1- *α*) and consequently exact confidence intervals are considered conservative [15].

#### Score confidence intervals

The score confidence interval [16] has its coverage close to the nominal confidence level even with small sample sizes. It has been shown to perform better than the Wald and the exact confidence intervals [1, 15]. The confidence limits for the *i*^*th*^study are computed as;

$$\begin{array}{l} \frac{p_{i}+\frac{z}{2n_{i}}\mp z\sqrt{p_{i}(1-p_{i})+\frac{\frac{z}{4n_{i}}}{n_{i}}}}{\left(1+\frac{z}{n_{i}}\right)}, \end{array}$$

where *z* is the ${\frac{\alpha }{2}}^{\text{th}}$ percentile of the standard normal distribution.

### Confidence Intervals for the pooled estimate after transformation

#### Freeman-Tukey double arcsine transformation

The variance stabilizing transformation of the proportions as proposed by Freeman and Tukey [12] normalizing the outcomes before pooling, is defined as;

$$\overset{-1}{\text{sin}}\sqrt{\frac{r_{i}}{n_{i}+1}}+\overset{-1}{\text{sin}}\sqrt{\frac{r_{i}+1}{n_{i}+1}}.$$

The asymptotic variance of the transformed variable is defined as, $\frac{1}{n_{i}+0.5}$. This transformations is intended to achieve approximate normality. The pooled estimate are then computed using the Dersimonian and Laird [6] method based on the transformed values and their variances. The confidence intervals for the pooled estimate are then computed using the Wald method.

#### Inverse of Freeman-Tukey double arcsine transformation

To convert the transformed values into the ‘original units’ of proportions, Miller [3] proposed the following formula;

$$\begin{array}{c} \;p=\frac{1}{2}\left[1-\text{sign}(\cos t)\sqrt{\left[1-{\left(\text{sin}t+\frac{\text{sin}t-\frac{1}{\text{sin}t}}{n}\right)}^{2}\right]}\right], \end{array}$$

where *t* is the transformed value and *n* is the sample size. In the meta-analysis setting, *t* is the pooled estimate or the confidence intervals based on transformed values. In practice, the use of this formula usually involves translating the means of *t*’s derived from binomials with different *n*’s as is the case in meta-analysis where most studies included have different sample sizes. In this case, Miller [3] suggested that the harmonic mean of the *n*_*i*_’s be used in the conversion formula. For a set of numbers, the harmonic mean is the inverse of the arithmetic mean of the reciprocals of the numbers in the set.

### The logistic-normal random-effects model

The observed events *r*_*i*_ are assumed to have a binomial distribution with parameters *p*_*i*_ and sample size *n*_*i*_, i.e;

$$r_{i}∼\text{binomial}(p_{i},n_{i}).$$

The normal distribution is then used to model the random-effects;

$$\text{logit}(p_{i})∼\text{normal}(\mu ,\tau ).$$

Here, *μ* is the mean of a population of possible means, and *τ* is the between-study variance, both in the logit scale. The maximum likelihood (ML) procedure is herein used to estimate *τ*. The above model can be reduced to form the fixed-effects model by assuming that *τ* = 0. In this case, the model is written as;

$$r_{i}∼\text{binomial}(p,n_{i}).$$

### Materials

The datasets used for the illustration were part of meta-analyses conducted by Arbyn et al. [17] and Dolman et al. [18]. The datasets are available as clickable examples in the help file for *metaprop*.

#### Dataset one

**Figure 1:**
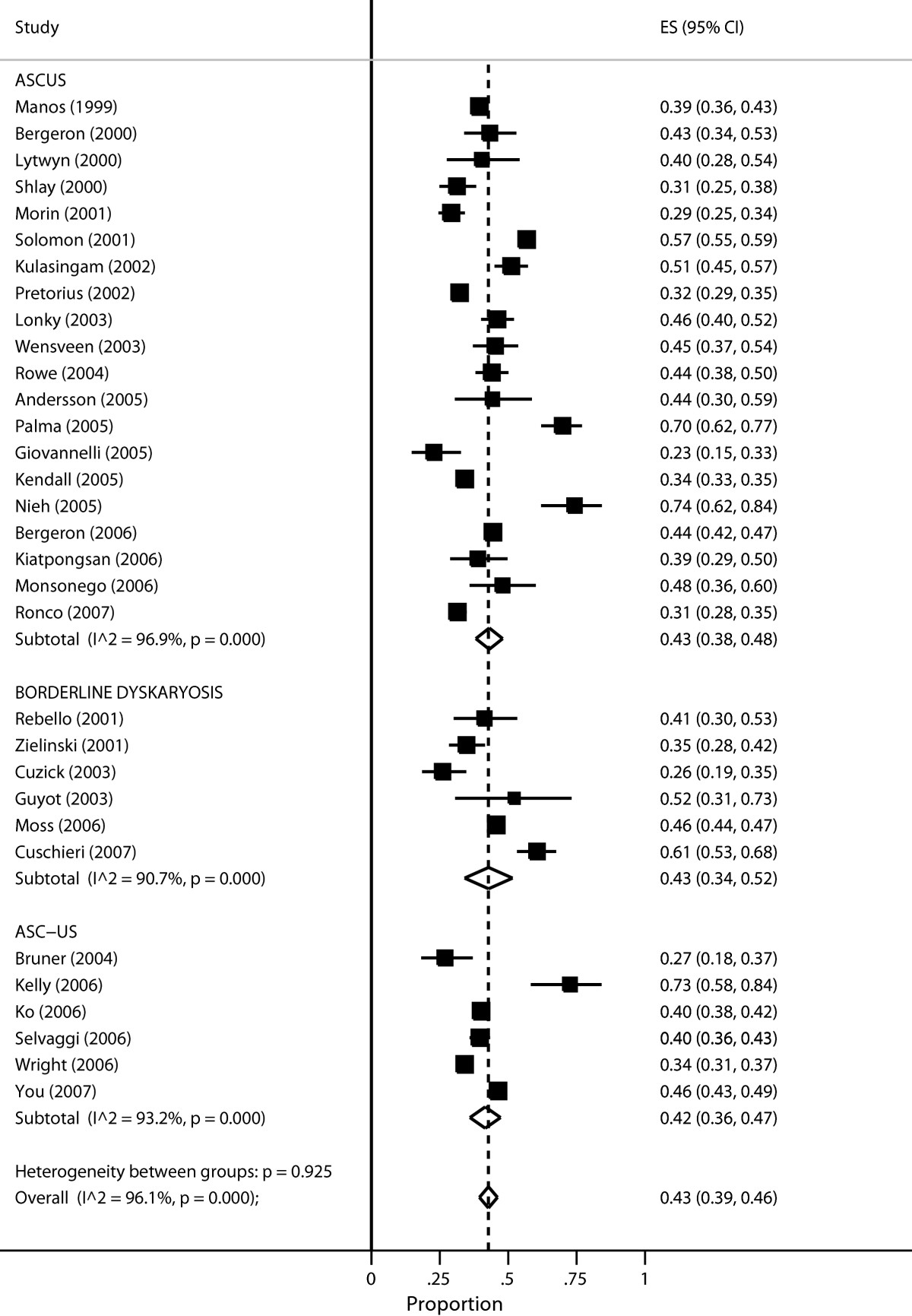
**Meta-analysis of the proportion of women with ASCUS or a borderline Pap smear that have a positive Hybrid Capture II test.** Output generated by the Stata procedure *metaprop*.

Arbyn et al. [17] assessed the HPV test positivity rate in women with equivocal or low-grade cervical cytological abnormalities. HPV testing has been proposed as a method to triage women with minor cytological abnormalities identified through screening for cervical cancer using the Pap smear [19, 20]. The prevalence of HPV infection reflects the burden of referral and diagnostic work-up when the test is used to triage women with these cytological conditions [17]. Two groups of minor cytological abnormalties can be distinguished: a) atypical squamous cells of undetermined significance (ASC-US) or borderline dyskaryosis and b) low-grade squamous intraepithelial lesion (LSIL) or mild dyskaryosis. The meta-analysis concluded that the large majority of women with LSIL were infected with HPV suggesting limited utility of HPV triaging. However, for women with ASC-US, more than halve tested negative and could be released from further follow-up. Figure 1 reproduces the meta analysis including 32 studies providing data of HPV infection in case of equivocal cervical cytology (ASC-US). The pooled prevalence of HPV infection, assessed with the Hybrid Capture 2 assay was 43% (95% CI: 39%-46%) (see Figure 1 and Table 2).

**Table 2:** **Meta-analysis of the presence of high-risk HPV DNA in women with equivocal cervical cytology, by terminology group (ASCUS, Borderline Dyskaryosis or ASC-US)**

Study	ES	[95% Conf. interval]		
**ASCUS**				
Manos (1999)	0.395	0.364	0.426	
Bergeron (2000)	0.432	0.339	0.53	
Lytwyn (2000)	0.404	0.276	0.542	
Shlay (2000)	0.313	0.248	0.383	
Morin (2001)	0.292	0.245	0.342	
Solomon (2001)	0.568	0.547	0.588	
Kulasingam (2002)	0.511	0.45	0.572	
Pretorius (2002)	0.322	0.293	0.353	
Lonky (2003)	0.46	0.401	0.521	
Wensveen (2003)	0.453	0.371	0.537	
Rowe (2004)	0.44	0.38	0.501	
Andersson (2005)	0.442	0.305	0.587	
Palma (2005)	0.699	0.62	0.769	
Giovannelli (2005)	0.228	0.147	0.328	
Kendall (2005)	0.341	0.33	0.352	
Nieh (2005)	0.742	0.62	0.842	
Bergeron (2006)	0.444	0.422	0.467	
Kiatpongsan (2006)	0.389	0.288	0.497	
Monsonego (2006)	0.479	0.359	0.601	
Ronco (2007)	0.314	0.281	0.349	
Sub-total				
Random pooled ES	0.431	0.382	0.480	
**BORDERLINE DYSKARYOS**				
Rebello (2001)	0.413	0.301	0.533	
Zielinski (2001)	0.347	0.284	0.415	
Cuzick (2003)	0.26	0.185	0.347	
Guyot (2003)	0.522	0.306	0.732	
Moss (2006)	0.456	0.44	0.473	
Cuschieri (2007)	0.605	0.532	0.675	
Sub-total				
Random pooled ES	0.428	0.341	0.516	
**ASC-US**				
Bruner (2004)	0.269	0.182	0.371	
Kelly (2006)	0.725	0.583	0.841	
Ko (2006)	0.401	0.381	0.421	
Selvaggi (2006)	0.396	0.359	0.434	
Wright (2006)	0.341	0.315	0.368	
You (2007)	0.463	0.434	0.492	
Sub-total				
Random pooled ES	0.416	0.360	0.472	
Overall				
Random pooled ES	0.428	0.395	0.461	
**Test(s) of heterogeneity:**				
	Heterogeneity statistic	Degrees of freedom	p-value	*I* ^2∗∗^
ASCUS	614.42	19	0.000	96.9%
BORDERLINE DYSKARYOS	53.58	5	0.000	90.7%
ASC-US	73.92	5	0.000	93.2%
Overall	785.77	31	0.000	96.1%
Random: Rest for heterogeneity between sub-groups:				
	0.16	2	0.925	
** *I* ^2^: the variation in ES attributable to heterogeneity				
Significance of test(s) of ES = 0				
	ASCUS	z = 17.22	p = 0.000	
	BORDERLINE			
	DYSKARYOS	z = 9.58	p = 0.000	
	ASC-US	z = 14.57	p = 0.000	
	Overall	z = 25.31	p = 0.000	

Output generated by the Stata procedure *metaprop*.

The dataset contains *author* and *year* which identify each study, where *tgroup* corresponds with the triage group(ASCUS, LSIL, borderline dyskaryosis). *num* and *denom* indicates the number of women with a positive HPV test (HC2 assay) and total number of tested women such that $\text{frac}\;\left(\frac{\text{num}}{\text{denom}}\right)$ is the proportion with a positive HC2 test. *se* indicates the standard error computed as $\sqrt{\frac{\text{frac}(1-\text{frac})}{\text{denom}}}$. *lo* and *up* are the lower and upper confidence intervals computed using the ‘exact’ method.

#### Dataset two

**Figure 2:**
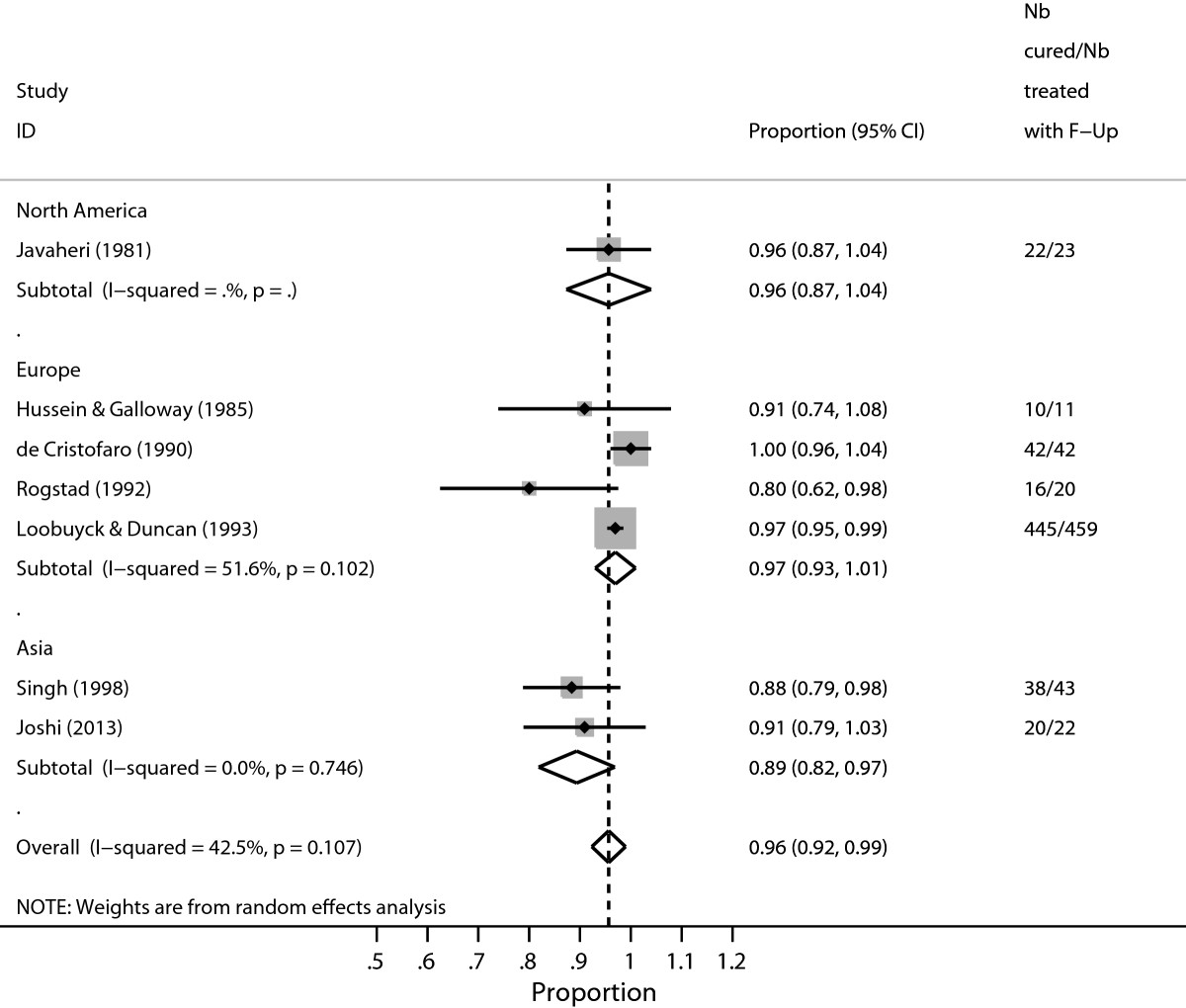
**Proportion-cured estimates associated with cold coagulation treatment for CIN1 disease, by world region as analysed by** ***metan*** **.**

Dolman et al. [18] published a systematic review on the efficacy of cold coagulation to treat cervical intraepithelial neoplasia (CIN). Thirteen reports were included in the meta-analysis which showed a high degree of heterogeneity among studies. Several studies had cure rates at or close to 100%. As seen in Figure 2, the Wald confidence intervals yield values beyond 1 for some of the individual studies and for the pooled proportion for studies conducted in Europe.

The dataset contains *nb_cured* and *nb_treated* indicates the number of women cured of CIN and total number of women treated for CIN such that $\text{frac}\;\left(\frac{\mathrm{nb}\_\text{cured}}{\mathrm{nb}\_\text{treated}}\right)$ is the proportion of women cured of CIN, and *se* is the standard error. *region* indicates continent in which the study was conducted. For studies with *frac* = 1, *se* = 0 and the authors replaced $\text{se}=\frac{up^{⌣}\text{low}}{2*1.96}$, where *up* and *low* were the exact binomial confidence intervals to ensure that such studies were not excluded from the analysis.

### Software development

The *metaprop* command is an adaptation of the metan programme developed by Harris et al. [10] intended to perform fixed and random-effects meta-analysis in Stata on continuous variables or associations between continuous or binomial variables. The *metaprop* program and its help file are available for downloading at http://ideas.repec.org/c/boc/bocode/s457781.html. The command requires Stata 10 or later versions and can be directly installed within Stata by typing *ssc install metaprop* when one is connected to the internet. An update to *metaprop* to include the logistic-normal random-effects model is also available for download. The updated command *metaprop_one* requires Stata 13 and can be directly installed within Stata by typing *ssc install metaprop_one* when one is connected to the internet.

## Results

### Example one

We reproduce Figure one in Arbyn et al. [17]. *metaprop* pools proportions and presents a weighted sub-group and overall pooled estimates with inverse-variance weights obtained from a random-effects model.

. metaprop num denom, random by(tgroup) cimethod(exact) /*

 */ label(namevar =author, yearvar =year) /*

 */ xlab(.25,0.5,.75,1)xline(0, lcolor(black)) /*

 */ subti(Atypical cervical cytology, size(4)) /*

 */ xtitle(Proportion,size(2)) nowt /*

 */ olineopt(lcolor(red)lpattern(shortdash))/*

 */ plotregion(icolor(ltbluishgray)) /*

 */ diamopt(lcolor(red)) /*

 */ pointopt(msymbol(x)msize(0))boxopt(msymbol(S) mcolor(black)) /*

Table 2 and Figure 1 both present the study specific proportions with 95% exact confidence intervals for each study, the sub-group and overall pooled estimate with 95% Wald confidence intervals and the *I*^2^ statistic which describes the percentage of total variation due to inter-study heterogeneity. The table presents additional information on the pooled proportions and includes tests of heterogeneity within the sub-groups and overall. Significant intra-group heterogeneity was observed (p <0.001 with *I*^2^ exceeding 93% for all the three terminology groups). However, no inter-group heterogeneity was noted (p = 0.925), supporting the pooling of all studies into one pooled measure: 43% (95% CI: 39-46%).

Though the weights have been computed using the random-effects model, the heterogeneity statistics have been computed by re-calculating the overall pooled estimate by treating the sub-group pooled estimates as though they were fixed-effects estimates. Since all study-specific proportions are close to 0.5, *metan* (see Figure one in Arbyn et al. [17]) and *metaprop* (see Figure 1) produce similar results.

### Example two

We extracted data that generated Figure two in Dolman et al. [18] (see Figure 2). Since the proportion of cured women is close to or at 1 in some studies, we enabled the Freeman-Tukey double arcsine transformation. Otherwise, studies with estimated proportion at 1 would be excluded from the analysis leading to a biased pooled estimate. Alternatively; using *cc(#)* ensures that such studies are not excluded. However, the pooled estimate is not guaranteed to be within the [0,1] interval which is automatic when the Freeman-Tukey double arcsine(*ftt*) option is enabled. We used the score confidence intervals for the individual studies.

. metaprop nb_cured nb_treated, random by(region)ftt cimethod(score)/* */label(namevar = study) graphregion(color(white)) plotregion(color(white))/* */ xlab(0.5,0.6,.7,0.8, 0.9, 1) /* */ xtick(0.5,0.6,.7,0.8, 0.9, 1) force/* */ xtitle(Proportion,size(2)) nowt stats /* */ olineopt(lcolor(black) lpattern(shortdash)) /* */ diamopt(lcolor(black)) /* */ boxopt(msymbol(S)) rcols(col)/* */ astext(70) texts(80) nohet notable

**Figure 3:**
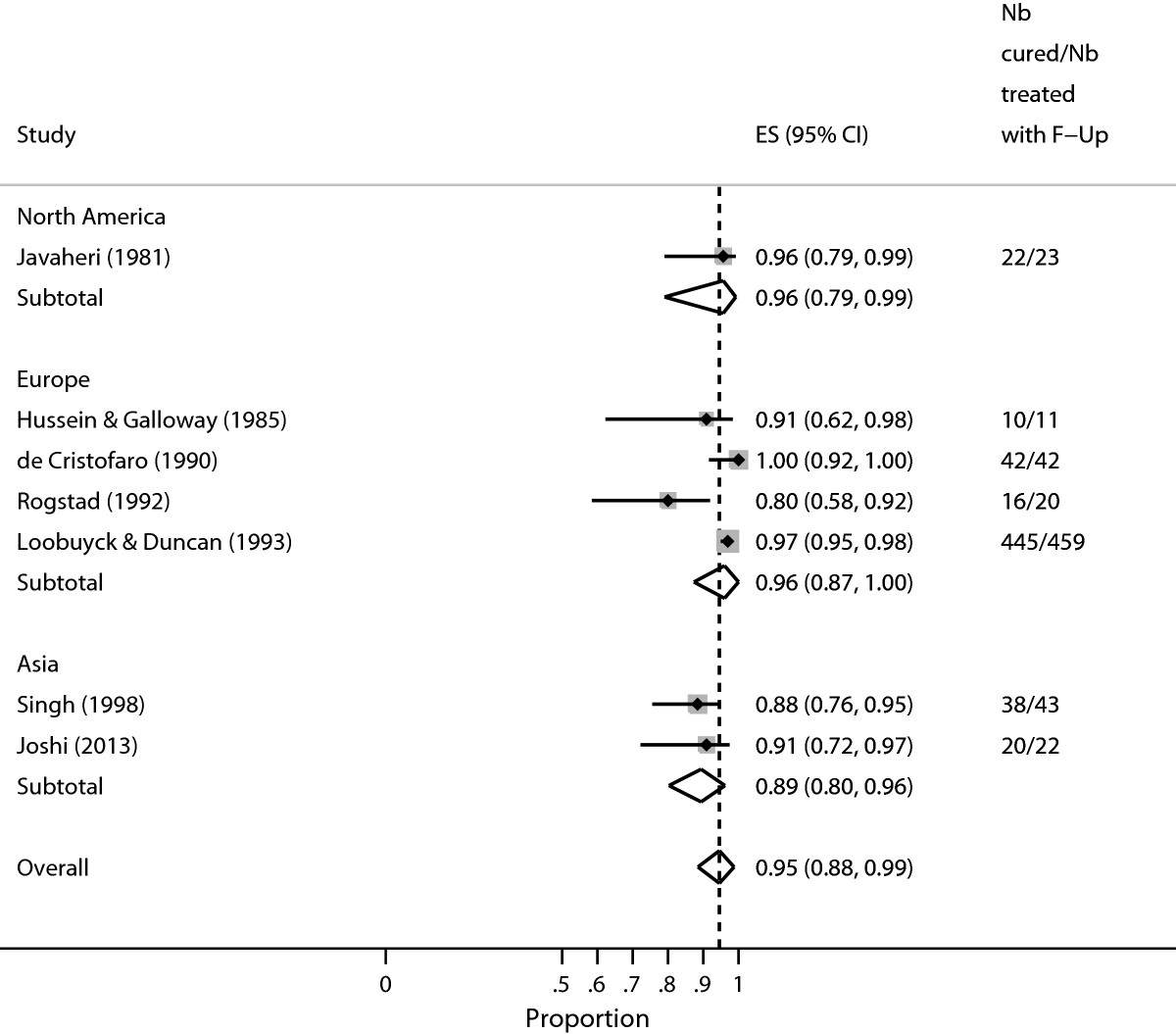
**Proportion-cured estimates associated with cold coagulation treatment for CIN1 disease, by world region as analysed by** ***metaprop*** **.**

Figure 3 (displaying the forest plot generated by *metaprop*) presents the study-specific proportions with 95% score confidence intervals, the regional and overall pooled estimates with 95% Wald confidence intervals, *I*^2^ statistic, and test of significance of the overall pooled estimates. In contrast with Figure 2 (displaying the graphical output generated with *metan*), all the confidence intervals have admissible values.

### Example three

We extracted data that generated Figure two in Dolmanet al. [18] (see Figure 2). We fit the logistic-normal random-effects model to the data. With these model, there is no worry about studies with cure rates close to or at 1 in some studies since we use the exact method. The confidence intervals for the individual studies also are computed with exact method. We used the updated command *metaprop_one* which requires Stata 13 to fit the generalized linear mixed model (GLMM).

. metaprop_one nb_cured nb_treated, random logit groupid(study) /// label(namevar =author, yearvar =year) sortby(year author) /// xlab(.1,.2,.3,.4,.5,.6,.7,.8,.9,1) xline(0, lcolor(black)) /// ti(Positivity of p16 immunostaining, size(4) color(blue)) /// subti("Cytology = HSIL", size(4) color(blue)) /// xtitle(Proportion,size(3)) nowt nostats /// olineopt(lcolor(red) lpattern(shortdash)) /// diamopt(lcolor(red)) pointopt(msymbol(s) msize(2)) /// astext(70) texts(100)

Table 3 presents the study-specific proportions with 95% exact confidence intervals and overall pooled estimates with 95% Wald confidence intervals with logit transformation and back transformation, *Chi*^2^ statistic of Likelihood ratio (LR) test comparing the random- and fixed-effects model, the estimated between-study variance and test of significance testing if the estimated proportion is equal to zero. The P-value for the LR is 0.022 indicating presence of significant heterogeneity. From the previous command, the Q-statistic is analogous to the LR statistic. In contrast with Figure 2 (displaying the graphical output generated with *metan*), all the confidence intervals have admissible values. The estimated pooled mean and the corresponding 95% intervals are similar to those obtained earlier (see Figure 2) computed as a weighted average after the arcsine transformation. However, the estimated between-study variance is larger (0.4907) than the Dersimonian and Laird variance estimate obtained from the previous command (0.0409) as expected [9].

**Table 3:** **Meta-analysis of the presence proportion of women cured of CIN1 disease with cold coagulation)**

Study		ES	[95% Conf. Interval]	
Javaheri (1981)		0.957	0.7901	0.9923
Hussein & Galloway (1985)		0.909	0.6226	0.9838
de Cristofaro (1990)		1.000	0.9162	1.0000
Rogstad (1992)		0.800	0.5840	0.9193
Loobuyck & Duncan (1993)		0.969	0.9495	0.9817
Singh (1998)		0.884	0.7552	0.9493
Joshi (2013)		0.909	0.7219	0.9747
Random pooled ES		0.942	0.8855	0.9715

LR test: RE vs FE Model *chi* ^2^ = 4.04 (d.f. = 1) p = 0.022. Estimate of between-study variance *Tau* ^2^ = 0.4907. Test of ES = 0 : z = 45.56 p = 0.000. Output generated by the Stata procedure *metaprop_one*.

## Discussion

We have presented procedures to perform meta-analysis of proportions in Stata. We adapted and made additions to the *metan* command to provide procedures which are specific for binomial data where the user specifies *n* and *N* denoting the number of individuals with the characteristic of interest and the total number of individuals. With *metaprop*, it is possible to perform a test of heterogeneity between groups when sub-group analysis is desired and the random-effects model has been used to compute the pooled estimate. In metan, a test for intergroup comparison is only produced when the fixed effects model is used in a subgroup meta-analysis.

When the estimated proportion is at 0/1, the estimate for the standard error is zero and therefore the Wald confidence intervals cannot be computed. Studies with zero standard error are often excluded since the weight assigned to such studies is infinite. Excluding such studies could lead to biased results and often users compute the standard error in ad hoc way. The continuity correction enabled by the cc(#) option avoids exclusion of studies with 0%. or 100% prevalence. While this ensures that the studies are retained, the confidence intervals for the pooled estimate may yield inadmissible values.

Furthermore, use of Wald confidence intervals for the individual studies when the estimated proportion is close to zero often yields inadmissible values. This is because the Wald confidence intervals are always symmetric around an estimate. In contrast to the Wald, the exact or score confidence intervals can be asymmetric especially near the extreme values. By computing the exact or score confidence intervals for the individuals studies, we are guaranteed of admissible values. While the exact confidence are regarded as the ‘gold’ standard, we recommend the use of score confidence intervals because the coverage is close to the nominal level, whereas the coverage is always higher than the nominal level for the exact method. By using the Freeman-Tukey double arcsine transformation, all the studies are retained, furthermore, we are guaranteed to have admissible confidence intervals for each individual study as well as for the pooled proportion. While the distribution of the Freeman-Tukey double arcsine statistic is more normal for sparse data, the procedure breaks down with extremely sparse data and should thus be used with caution [21]. Whenever possible the use of exact methods is more recommended for binomial data. As the sample size increases and when the proportions are not extreme, methods relying on transformed data and exact methods give similar results as approximate methods.

## Conclusion

*metaprop* enables epidemiologists to pool proportions in Stata, avoiding problems encountered with *metan*. *metaprop* allows inclusion of studies with proportions equal to zero or 100 percent, and avoids confidence intervals exceeding the 0 to 1 range. The logistic-normal random-effects model draws the users a step closer towards the use of exact methods recommended for binomial data.

## Authors’ original submitted files for images

Below are the links to the authors’ original submitted files for images. Authors’ original file for figure 1 Authors’ original file for figure 2 Authors’ original file for figure 3
