# Supplementary figures and images for: Metaprop: a Stata command to perform meta-analysis of binomial data
Source: Arch Public Health. 2014 Nov 10;72:39. doi: 10.1186/2049-3258-72-39 (PMC4373114; doi:10.1186/2049-3258-72-39)

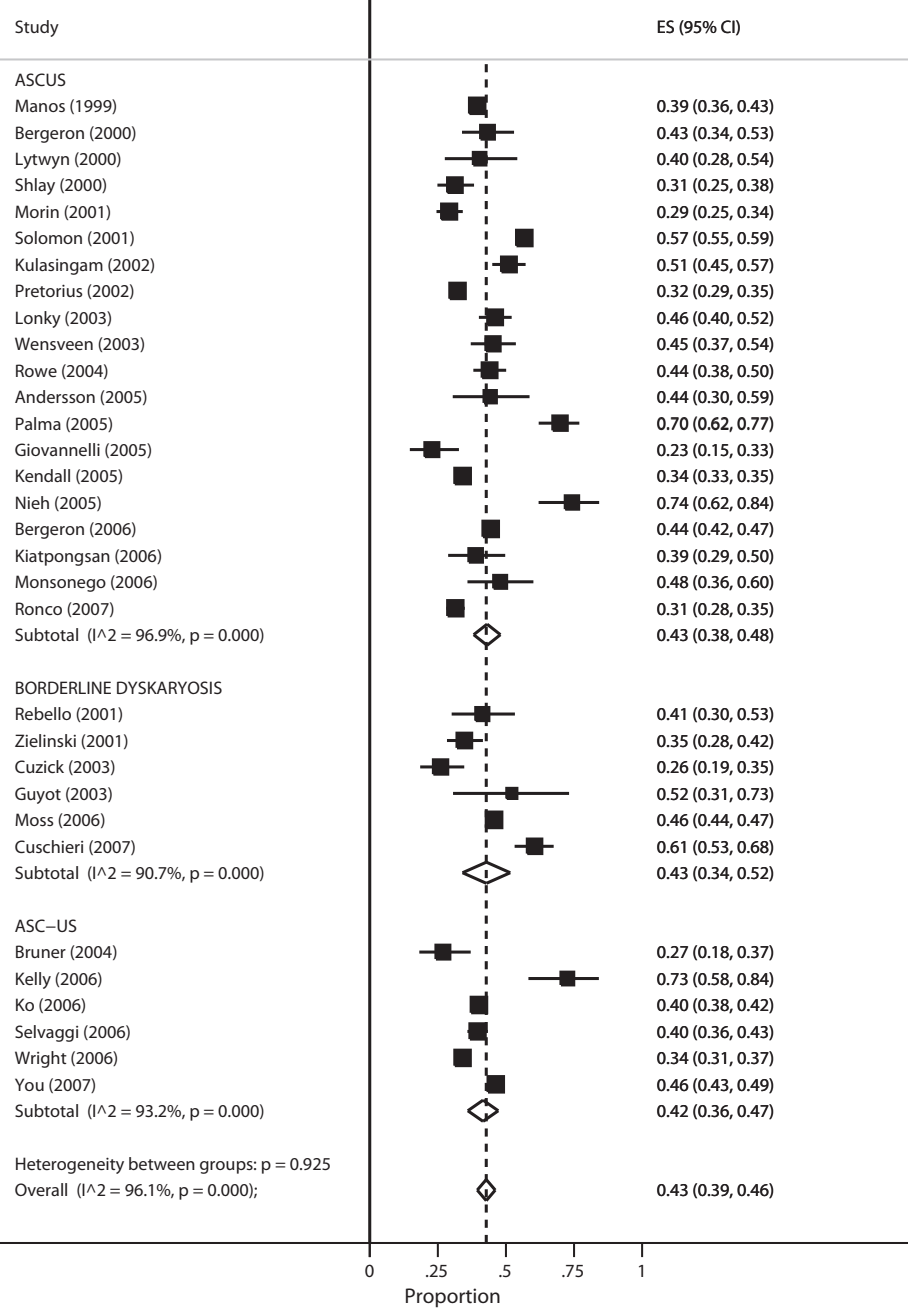

Supplement: Supplementary file 1 — Authors’ original file for figure 1 [file 13690_2014_5060_MOESM1_ESM.pdf]

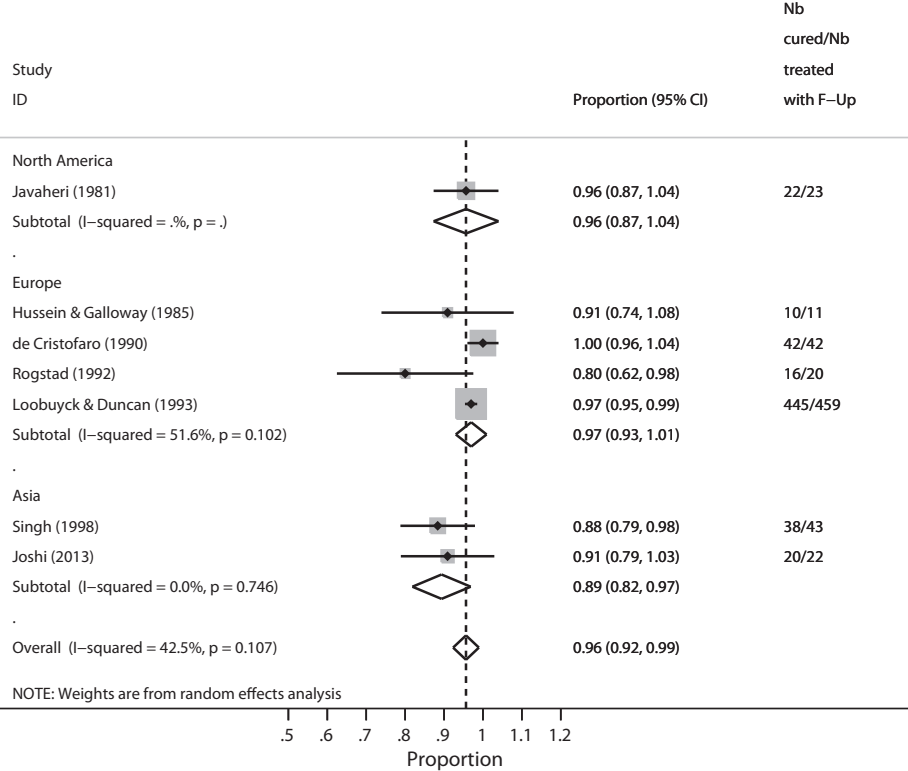

Supplement: Supplementary file 2 — Authors’ original file for figure 2 [file 13690_2014_5060_MOESM2_ESM.pdf]

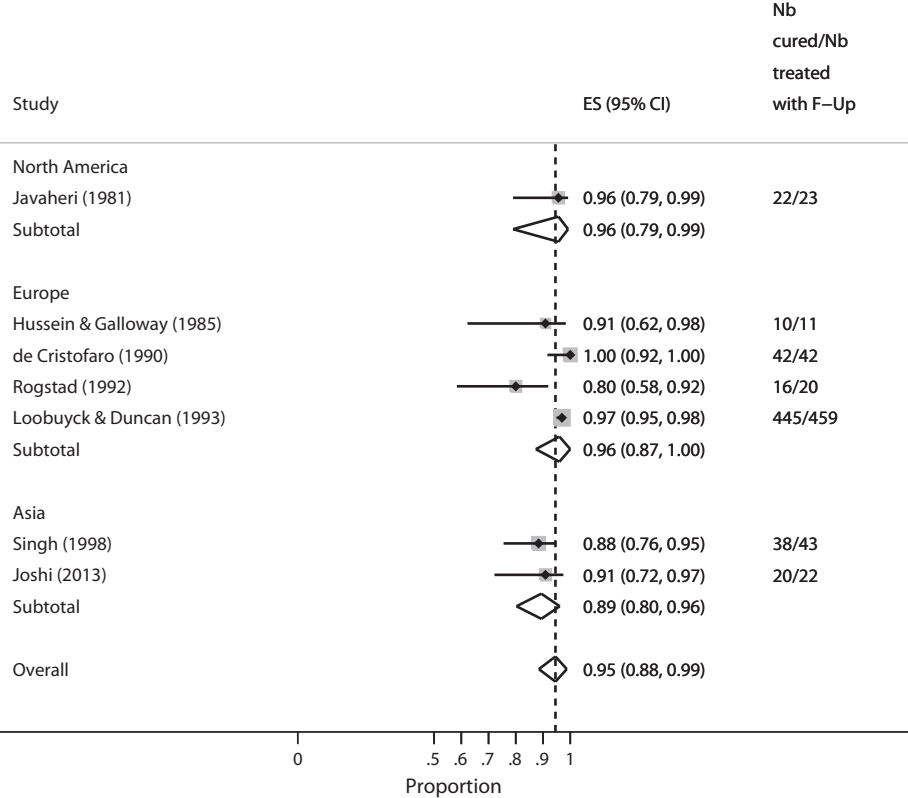

Supplement: Supplementary file 3 — Authors’ original file for figure 3 [file 13690_2014_5060_MOESM3_ESM.pdf]
